# Pharmacokinetics of quinacrine in the treatment of prion disease

**DOI:** 10.1186/1471-2334-4-53

**Published:** 2004-11-29

**Authors:** Lotus Yung, Yong Huang, Pierre Lessard, Giuseppe Legname, Emil T Lin, Michael Baldwin, Stanley B Prusiner, Chongsuk Ryou, B Joseph Guglielmo

**Affiliations:** 1 Department of Clinical Pharmacy, School of Pharmacy, University of California San Francisco, San Francisco, California 94143-0622, USA; 2 Drug Studies Unit, Department of Biopharmaceutical Sciences, School of Pharmacy, University of California San Francisco, San Francisco, California 94143-0446, USA; 3 Department of Pharmaceutical Chemistry, School of Pharmacy, University of California San Francisco, San Francisco, California 94143-0446, USA; 4 Department of Neurology, School of Medicine, University of California San Francisco, San Francisco, California 94143-0114, USA; 5 Department of Biochemistry and Biophysics, School of Medicine, University of California San Francisco, San Francisco, California 94143-0448, USA; 6 Institute for Neurodegenerative Diseases, University of California, San Francisco, San Francisco, California 94143-0518, USA

## Abstract

**Background:**

Prion diseases are caused by the accumulation of an aberrantly folded isoform of the prion protein, designated PrP^Sc^. In a cell-based assay, quinacrine inhibits the conversion of normal host prion protein (PrP^C^) to PrP^Sc ^at a half-maximal concentration of 300 nM. While these data suggest that quinacrine may be beneficial in the treatment of prion disease, its penetration into brain tissue has not been extensively studied. If quinacrine penetrates brain tissue in concentrations exceeding that demonstrated for *in vitro *inhibition of PrP^Sc^, it may be useful in the treatment of prion disease.

**Methods:**

Oral quinacrine at doses of 37.5 mg/kg/D and 75 mg/kg/D was administered to mice for 4 consecutive weeks. Plasma and tissue (brain, liver, spleen) samples were taken over 8 weeks: 4 weeks with treatment, and 4 weeks after treatment ended.

**Results:**

Quinacrine was demonstrated to penetrate rapidly into brain tissue, achieving concentrations up to 1500 ng/g, which is several-fold greater than that demonstrated to inhibit formation of PrP^Sc ^in cell culture. Particularly extensive distribution was observed in spleen (maximum of 100 μg/g) and liver (maximum of 400 μg/g) tissue.

**Conclusions:**

The documented extensive brain tissue penetration is encouraging suggesting quinacrine might be useful in the treatment of prion disease. However, further clarification of the distribution of both intracellular and extracellular unbound quinacrine is needed. The relative importance of free quinacrine in these compartments upon the conversion of normal host prion protein (PrP^C^) to PrP^Sc ^will be critical toward its potential benefit.

## Background

Prion diseases, while rare, invariably result in fatal neurodegeneration. At present, no therapy has been proven to be useful in the treatment of prion disease. However, acridine and phenothiazine derivatives have been evaluated [1,2] using an *in vitro *model, and quinacrine and chlorpromazine inhibit PrP^Sc ^formation in a scrapie-infected neuroblastoma (ScN2a) cell line [2]. Half-maximal inhibition of PrP^Sc ^formation at effective concentrations (EC_50_) for quinacrine was found to be 300 nM (120 μg/ml) [2]. While this concentration represents the concentration of quinacrine added to the cell culture, it does not reflect the effective intracellular quinacrine concentrations. In the past, quinacrine was used as an antiparasitic agent in the treatment of malaria and giardiasis, however, more effective, less toxic agents have since replaced this agent [3]. While quinacrine may be useful in the treatment of prion disease, its pharmacokinetics have not been extensively studied. Studies from the 1940s suggest that quinacrine is associated with extensive tissue distribution and a prolonged pharmacologic half-life [3,4]. However, the rate and extent of quinacrine penetration into brain tissue has not been characterized. If quinacrine administration *in vivo *results in brain tissue concentrations exceeding that shown to inhibit PrP^Sc ^*in vitro*, it may constitute an effective therapy for prion disease. While the precise site of antiprion action is unknown (intracellular versus extracellular) adequate quinacrine penetration into brain tissue might result in an effective agent for the treatment of prion diseases.

The objective of this study was to characterize the achievable plasma concentrations and associated tissue (brain, liver, spleen) penetration associated with quinacrine.

## Methods

### Animal Model

The protocol was approved by the institutional animal care and use committee. The Animal Facility of the Institute for Neurodegenerative Diseases provided animal samples from 2 different strains of mice, FVB and CD1. Twenty four animals of each strain (24 FVB and 24 CD1 strain animals) were fed 37.5 mg/kg/D and 24 animals of each strain were fed 75 mg/kg/D of oral quinacrine over a 4-week period (weeks 1–4), given *ad lib *as a chocolate flavored liquid diet. From weeks 5–8 animals were given regular feed without quinacrine. At weekly intervals, 3 animals of each strain were euthanized and tissues were collected for analysis.

### Quinacrine level analysis

#### Preparation of standard solutions

Quinacrine dihydrochloride (purity: 98.6%) was purchased from Fluka, while sulfadimethoxine sodium salt (purity: 98%) which served as the internal standard (IS) was obtained from Sigma. Quinacrine stock solution was prepared as 1 mg/ml in methanol. Sulfadimethoxine stock solution was prepared as 1 mg/ml in 50% methanol. The working solutions were prepared by diluting the respective standard and control stock solutions with 50% methanol and 0.1% formic acid to 2 μg/ml and 100 ng/ml, respectively. All solutions were stored in a 4°C refrigerator in silinized brown glass containers.

#### LC/MS/MS system and conditions

The HPLC system employed a Shimadzu LC-10 AD pump and a Waters intelligent Sample Processor 717 Plus autosampler/injector. A BDS Hypersil C18 column, 50 × 4.60 mm, 5 μm particle size was directly coupled to a Micromass Quattro LC Ultima triple quadrupole tandem mass spectrometer using electrospray ionization in positive ion mode. The sample cone voltage and collision energy were 25 V and 20 eV respectively for both quinacrine and the internal standard and the source block and desolvation temperature were 100°C and 400°C, respectively. The mass scanning mode employed multiple reaction monitoring (MRM) with the singly charged quinacrine ion selected at m/z 400.5 giving a fragment ion at m/z/142.0, and the internal standard at m/z 311.0→156.0. The mobile phase consisted of CH_3_OH/H_2_O/TFA (45:55:0.05) with 1 mM ammonium formate. The flow rate was 0.8 ml/min with 1/4 split into the mass spectrometer. The injection volume was 5–10 μl with a run time of 3.5 min.

### Sample preparation

All samples were stored at -70°C until analyzed. Each tissue sample was subjected to a specific method, as described below, for drug extraction and for the determination of concentration. All samples were analyzed by LC/MS/MS.

### Accuracy and precision

Accuracy and precision was demonstrated throughout the working range with interday and intraday coefficient of variation and relative error <10%.

### Plasma extraction

Each plasma sample was thawed at room temperature for 10–15 min; then 20 μl of plasma was aliquotted to a new test tube. To each tube 200 μl of 70% acetonitrile solution, containing 0.1% formic acid and 50 ng/mL of internal standard, was added. The test tubes were vortexed at high speed for 1 min and centrifuged at 10,000 rpm for 10 min. The supernatant was transferred into the autosampler for LC/MS/MS analysis. A set of standard curves with a duplicate set of quality control (QC) samples was generated for sample analysis.

### Brain samples

Prior to the in vivo study, the stability of quinacrine in mouse brain tissue was determined. Using 3 different doses of quinacrine (4 mg/kg/d, 80 mg/kg/d, 160 mg/kg/d), mouse brain tissue was soaked in 100% methanol for 7 days. Samples were taken on days 0,1,2,3, and 7 to analyze quinacrine concentrations using LC/MS/MS. Complete equilibration was observed by Day 1 and no degradation in quinacrine was noted through day 7.

Brain samples were thawed (in plastic tubes) at room temperature for 15–20 min and weighed. To each sample 0.5 ml of 0.9% NaCl was added followed by incubation at room temperature for 1 h. For the standard curve and quality control samples, a brain tissue sample from an untreated mouse was spiked with different amounts of quinacrine and incubated at room temperature for 1 h. After the addition of 100 μl of 1 μg/ml internal standard solution in 50% methanol and 0.1% formic acid, 5 ml of 100% methanol was added into each sample and soaked at 4°C for two weeks. On the last day, 200 μl was aliquotted from each brain sample and placed into the autosampler for analysis via LC/MS/MS.

### Liver samples

Liver samples were thawed at room temperature for 15–30 min and weighed. Considering the increased size of the liver samples and that the increased connective tissue in liver samples could potentially prevent the complete distribution of quinacrine into methanol, homogenization was used for these samples. To each sample 100% methanol was added (10 ml/g of tissue) and the tissue was homogenized in ice water for 1 min at speed 3 (Tissue Tearor, model 985-370, Biospec Products, Inc). The internal standard (100 μl of 10 μg/ml in 50% methanol, 0.1% formic acid) was added to 0.2 ml of each homogenized liver sample in a glass test tube. Samples were vortexed for 1 min, centrifuged at 3000 rpm for 10 min and 20 μl of each supernatant was transferred into a new test tube. Each sample was further diluted with 4 ml of 50% methanol, vortexed, and 200 μl was transferred to the autosampler for LC/MS/MS analysis. A set of standard curve with a duplicate set of QC samples was generated for sample analysis.

### Spleen samples

Spleen sample preparation was similar to the preparation of brain samples using a methanol soak. Prior to the in vivo study, the stability of quinacrine in mouse spleen tissue was determined. Using 3 different doses of quinacrine (4 mg/kg/d, 80 mg/kg/d, 160 mg/kg/d), mouse spleen tissue was soaked in 100% methanol for 7 days. Samples were taken on days 0, 1, 2, 3, and 7 to analyze quinacrine concentrations using LC/MS/MS. Complete equilibration took place by Day 1 and no degradation in quinacrine was observed through day 7.

Samples from the in vivo analysis were soaked for 14 days at 4°C. On day 14, 50 μl was aliquotted from each spleen sample and diluted with 1 ml of 50% methanol. Each sample was vortexed for 1 min and 200 μl was transferred to the autosampler for LC/MS/MS analysis. A set of standard curves with a duplicate set of QC samples was generated for sample analysis.

## Results

### Quinacrine analysis

**Figure 1:**
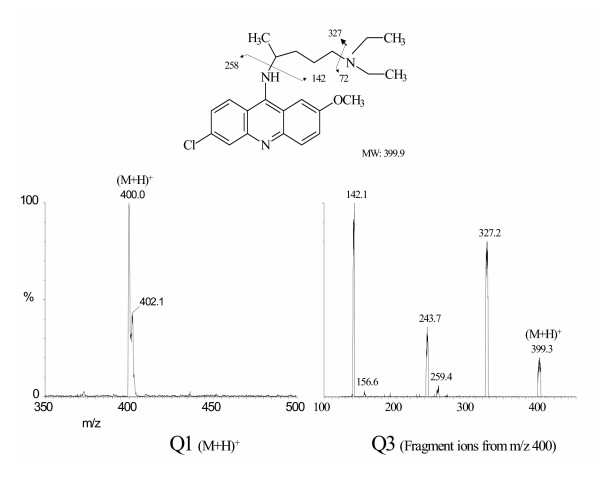
Q1 and Q3 mass spectra of quinacrine

**Figure 2:**
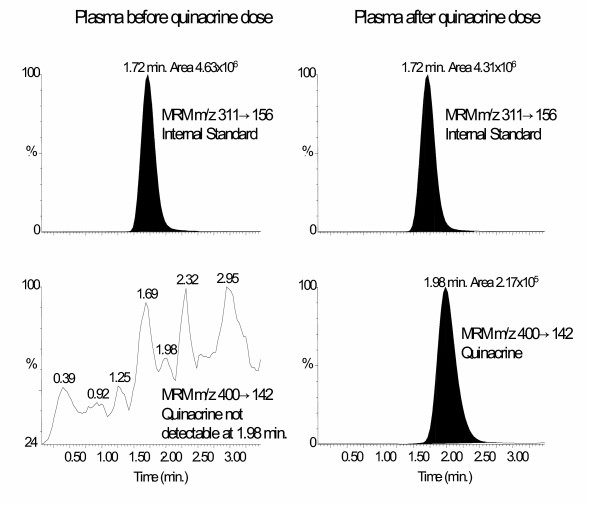
LC/MS/MS chromatograms of quinacrine in mouse plasma

**Figure 3:**
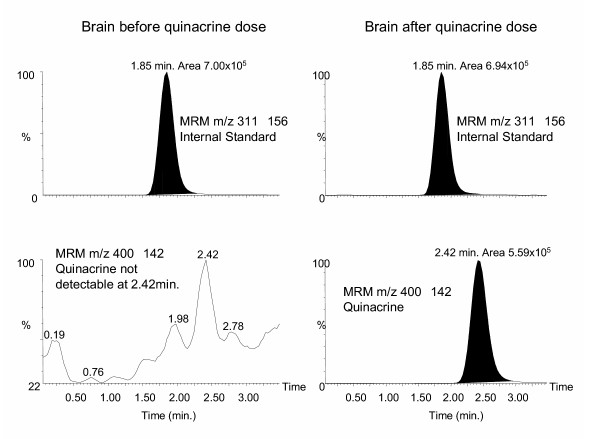
LC/MS/MS chromatograms of quinacrine in mouse brain

The mass spectrum of quinacrine (Q1) and its tandem spectrum (Q3), showing the fragment ion selected for MRM, are shown in Figure 1. Using MRM for m/z 400.5 ± 142.0 and the appropriate LC/MS/MS conditions described above, quinacrine could be selectively and sensitively detected in plasma (Fig. 2) and brain tissue (Fig. 3) after relatively simple sample preparation.

### In vivo studies

In plasma, quinacrine concentrations with the 37.5 mg/kg/D dose ranged from 75 to 175 ng/ml for both FVB and CD1 strains mice and remained at this level during the 4-week dosing interval. Once discontinued, quinacrine was completely eliminated from plasma, with no drug detectable within one week. At 75 mg/kg/D quinacrine, plasma concentrations reached >2 × those observed with the lower dose, averaging 300 to 400 ng/ml.

**Figure 4:**
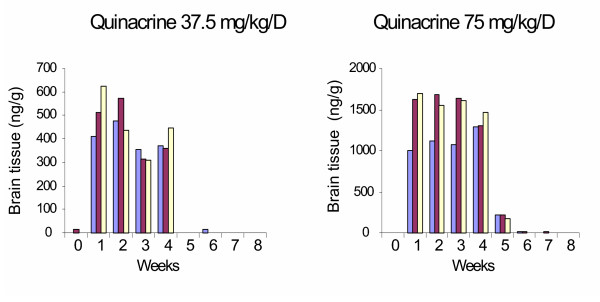
Quinacrine brain levels in mice (FVB strain) administered oral quinacrine at 37.5 or 75 mg/kg/D for 4 weeks

Quinacrine levels in brain tissue for both mice strains were determined to be substantially greater than those achieved in plasma. Similar to observations with plasma, steady-state levels in the brain with the 37.5 mg/kg/D dose were achieved by the end of the first week, averaging 400 to 600 ng/g brain tissue. Figure 4 characterizes the brain tissue levels in the FBV mice. Quinacrine was undetectable in brain tissue within a week after discontinuing treatment. With the 75 mg/kg/D dose, quinacrine levels in brain tissue were observed to be greater than 2 × those observed with the 37.5 mg/kg/D dose, averaging 1500 ng/g. After high-dose (75 mg/Kg/D) treatment ceased, quinacrine levels in brain were detected for two weeks.

**Figure 5:**
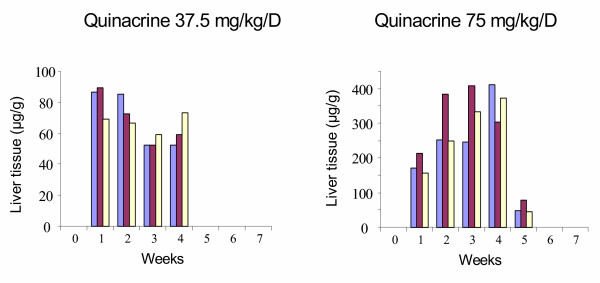
Quinacrine liver levels in mice (FVB strain) administered oral quinacrine at 37.5 or 75 mg/kg/D for 4 weeks

**Figure 6:**
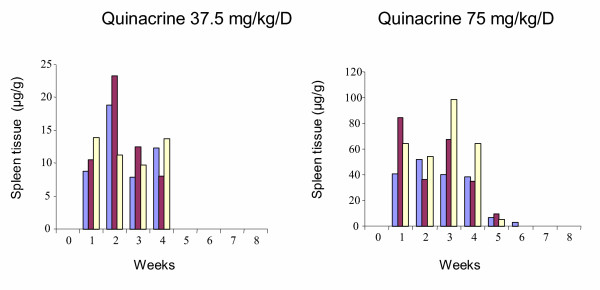
Quinacrine spleen levels in mice (FVB strain) administered oral quinacrine at 37.5 or 75 mg/kg/D for 4 weeks

The analysis of liver samples showed particularly high quinacrine levels, at many times those observed in plasma and brain. At the end of the first week of treatment with 37.5 mg/kg/D, steady-state levels of 70 to 90 μg/g were achieved in liver tissue in both mice strains, which remained throughout the course of treatment. Figure 5 demonstrates the achievable liver tissue levels in FBV mice. In contrast, the 75 mg/kg/D dosing resulted in gradually increasing levels in liver tissue, rising from approximately 150 μg/g in the first week to a steady-state level of 300 to 400 μg/g by the end of the 4^th ^week. Similarly, spleen tissue levels in both mice strains were very elevated, averaging 5 to 10 μg/g with the 37.5 mg/kg/D dose and 40 to 100 μg/g with the 75 mg/kg/D dose. Figure 6 characterizes the spleen tissue levels of the FBV mice.

Of note, in contrast with plasma and brain tissue, in which dose dependency was somewhat linear, increasing quinacrine doses in spleen and liver were associated with substantially greater tissue levels. In addition to the isolation of quinacrine, numerous metabolites were identified in all tested tissue samples (data not shown).

## Discussion

The current study is the first comprehensive evaluation of quinacrine distribution in plasma and brain tissue. Shannon et al. evaluated the pharmacokinetics of quinacrine in the treatment of malaria [3]. Using a dog model, these researchers observed quinacrine to be concentrated in the liver and spleen, as well as muscle and lung. In the same study, humans receiving 100 mg quinacrine three times daily achieved maximal plasma concentrations of 100 ng/ml. Administration of a single intravenous dose of 2 mg/kg quinacrine in rabbits was associated with plasma levels of 10 ng/ml [4]. The quinacrine levels we observed in plasma are similar to those recorded from oral dosing for malaria [3]. We observed rapid and extensive distribution of quinacrine in brain, liver, and spleen tissue in association with much lower plasma concentrations. Although quinacrine was consistently observed one week after drug discontinuation in all tissue at the 75 mg/kg/D dose, none could be observed one week after cessation of the 37.5 mg/kg/D doses.

The concentrations we report in brain in terms of concentration/gm of tissue are several-fold greater than that shown for effective antiprion activity in ScN2a cells [2]. In that *in vitro *study, the EC_50 _for quinacrine was 300 nM, which approximates 120 ng/ml. We report here that quinacrine levels in brain tissue averaged 400 to 600 ng/g with the 37.5 mg/kg/D dose and 1500 ng/g with the 75 mg/kg/D dose, concentrations that exceed the *in vitro *EC_50 _by 3- to 10-fold.

Others have reported the antiprion function of quinacrine *in vitro*, including clearance of PrP^Sc ^(5) and/or inhibition of PrP^Sc ^formation [6]. Barret et al., who also noted the *in vitro *benefit from quinacrine, found the drug to be ineffective in the animal model under the conditions employed [1]. In contrast, some case reports in humans suggest quinacrine to be associated with clinical improvement, including the return of voluntary eye movement [7,8].

While the current study has confirmed brain tissue concentrations in excess of the reported EC_50_, it important to note critical limitations in their interpretation. Quinacrine has been determined to be highly protein-bound [3], measured at 83–90% in older studies. Additionally, the drug has been shown to be highly concentrated in white blood cells with intracellular levels ranging from 9,500–18,400 μg/L with accompanying low CSF levels (4.3–5.4 μg/L). Previous investigations confirm that it is free drug that is microbiologically active in the treatment of infection [9]. The brain tissue levels in the current investigation represent the sum total of intracellular, extracellular, protein-bound and unbound quinacrine. Highly protein-bound agents penetrate less well between plasma and tissue compartments, suggesting that quinacrine would more likely be plasma-bound. However, the results of our study strongly suggest deep brain tissue penetration of quinacrine, suggesting the possible contribution of membrane transporter proteins or other mechanisms facilitating passage of quinacrine into brain tissue. Considering the extensive intracellular white blood cell concentrations achieved with quinacrine, similar mechanisms may be responsible in actively pumping quinacrine into these cells. Consequently, it is critical to determine the actual free quinacrine concentrations in both intracellular and extracellular brain tissue and to document the relative contribution of these compartments in the pathogenesis and treatment of prion disease. Using microdialysis probes, Mindermann and colleagues [10] evaluated the penetration of rifampin into cerebral extracellular space, brain tumor, perifocal, and normal brain tissue. The findings confirmed consistent cerebral extracellular space concentrations, but remarkably different rifampin brain tissue levels, particularly concentrating in brain tumor tissue. Considering the pathogenesis of prion disease, heterogeneity of brain tissue concentrations may impact similarly the efficacy of quinacrine or other agents. Our results strongly suggest that similar microdialysis experiments take place with quinacrine to clarify the distribution characteristics of this agent.

In addition to steady-state levels of quinacrine, we observed a number of quinacrine metabolites in all tissue samples studied, which raises the possibility that these metabolites may have *in vitro *antiprion activity similar to or greater than that of the parent compound. These tests warrant further evaluation.

## Conclusions

Based upon its oral bioavailability, favorable tissue distribution characteristics, and *in vitro *activity, quinacrine may be a useful agent in the treatment of prion disease. However, more detailed tissue distribution analyses linked with the critical sites of prion action are warranted.

## Competing interests

The author(s) declare that they have no competing interests.

## Authors' contributions

LY, YH, ETL developed the assay, performed sample analyses, contributed to the writing of the paper. PL, GL, MB, CR developed the protocol and performed the animal model experiments. SBP conceived of the study and participated in its coordination. BJG drafted the manuscript, participated in its coordination and mentored LY. All authors read and approved the final manuscript.

## Pre-publication history

The pre-publication history for this paper can be accessed here:
